# MAGPEL: an autoMated pipeline for inferring vAriant-driven Gene PanEls from the full-length biomedical literature

**DOI:** 10.1038/s41598-020-68649-0

**Published:** 2020-07-23

**Authors:** Nafiseh Saberian, Adib Shafi, Azam Peyvandipour, Sorin Draghici

**Affiliations:** 1 Department of Computer Science, Wayne State University, Detroit, MI USA; 2 Department of Obstetrics and Gynecology, Wayne State University, Detroit, MI USA

**Keywords:** Literature mining, Machine learning

## Abstract

In spite of the efforts in developing and maintaining accurate variant databases, a large number of disease-associated variants are still hidden in the biomedical literature. Curation of the biomedical literature in an effort to extract this information is a challenging task due to: (i) the complexity of natural language processing, (ii) inconsistent use of standard recommendations for variant description, and (iii) the lack of clarity and consistency in describing the variant-genotype-phenotype associations in the biomedical literature. In this article, we employ text mining and word cloud analysis techniques to address these challenges. The proposed framework extracts the variant-gene-disease associations from the full-length biomedical literature and designs an evidence-based variant-driven gene panel for a given condition. We validate the identified genes by showing their diagnostic abilities to predict the patients’ clinical outcome on several independent validation cohorts. As representative examples, we present our results for acute myeloid leukemia (AML), breast cancer and prostate cancer. We compare these panels with other variant-driven gene panels obtained from Clinvar, Mastermind and others from literature, as well as with a panel identified with a classical differentially expressed genes (DEGs) approach. The results show that the panels obtained by the proposed framework yield better results than the other gene panels currently available in the literature.

## Introduction

One crucial step in understanding the biological mechanism underlying a disease condition is to capture the relationship between the variants and the disease risk^1^. There are several publicly available databases contain the disease-associated variants such as Clinvar^2^, SNPedia^3^, OMIM^4^, Swiss-Prot^5^, COSMIC^6^, BioMuta^7^, HGMD^8^, UMD^9^, HGVbaseG2P^10^, MutDB^11^, dbSNP^12^, PharmGKB^13^ and InSiGHT^14^. All these databases are manually curated by human experts. While this manual curation ensures a high quality of the annotations, the manual extraction of this type of information from the biomedical literature takes an enormous amount of time and effort. The current rate with which new variants are published is simply too high for any manual annotation process. As an additional challenge, despite the HGVS (Human Genome Variation Society) standard recommendations for the description of the variants, many variants are still reported in literature in non-standard formats. A number of automatic mutation indexing tools have been developed. Such tools process biomedical literature and produce a list of mutations that appear in these papers. These include MutationFinder^15^, EMU^16^, MEMA^17^, MuteXt^18^, Mutation GraB^19^ and MutationMiner^20^. The most recent such tool, tmVar 2.0^21^ extracts variants from an article and normalizes them to their unique dbSNP identifiers. The next step is to develop software tools to extract variants-disease associations from the biomedical literature. Several methods have been proposed for this purpose such as MuGeX^22^, OSIRIS^23^, EnzyMiner^24^ and the methods proposed by Singhal et al.^1,25^. All these methods have been applied to only the title and the abstract section of biomedical articles. However, a comprehensive study showed that a significant number of genetic variants are only included in the full text and the supplementary materials of the articles^26^. These will be missed if the variants are only extracted from titles and abstracts. Doughty et al.^16^ also proposed a tool named EMU for extracting the disease-associated mutations from biomedical literature. Although this tool automatically extracts the mutations and their corresponding genes from an article, it still requires human curation to discover the mutation-disease associations.

Here we propose an automated framework to extract disease-associated variants from the full-length biomedical literature and design a variant-driven gene panel for a given disease phenotype. As the first step, the proposed framework employs word cloud analysis to identify the variant-relevant articles. The variant-gene-disease associations are then extracted from these articles. An evidence-based variant-driven gene panel is then generated based on the mined triplet information. A comprehensive validation procedure illustrates the capabilities of the proposed framework. We validate the proposed variant-driven gene panels by showing their abilities to predict the patients’ clinical conditions (healthy vs. disease) on multiple independent validation datasets.

## Methods

**Figure 1:**
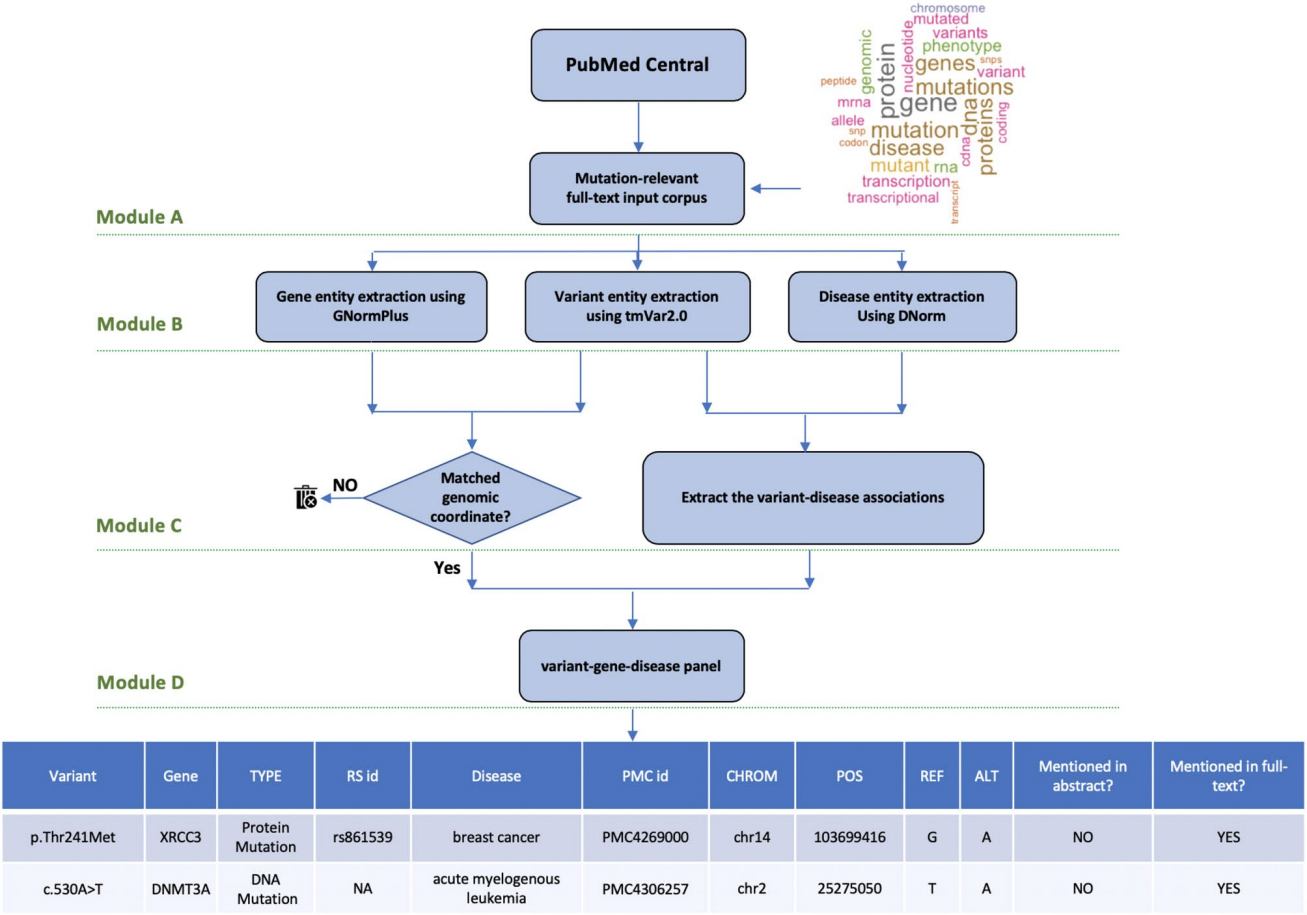
Framework overview. Module (**A**) obtains all the publicly available full-length articles from the PubMed Central (PMC) database. Then it uses the word cloud analysis and generate a weighted list of variant-relevant keywords. The variant-relevant articles are then selected based on the presence of this list in their full text (“Variant-relevant input corpus”). Module (**B**) uses GNormPlus^27^, tmVar 2.0^21^ and DNorm^28^ tools to extract the gene, variant and disease phenotype entities, respectively (“Extract the variant, gene and disease entities”). Module (**C**) extracts the gene-variant associations from each input article (“Extract the variant-gene associations”). This module also uses a set of features to discover the disease-variant associations (“Extract the variant-disease associations”). Module (**D**) generates a panel consists of the variant-gene-disease associations.

Figure 1 illustrates the proposed framework that consists of the following four major modules: (1) obtain the full-length variant-relevant articles; (2) extract all the variant, gene and disease entities from each input article; (3) identify the variant-gene and the variant-disease associations in each input article; (4) design a variant-driven gene panel for a given phenotype. The detailed descriptions of each step are provided in the following sections.

### Variant-relevant input corpus

**Figure 2:**
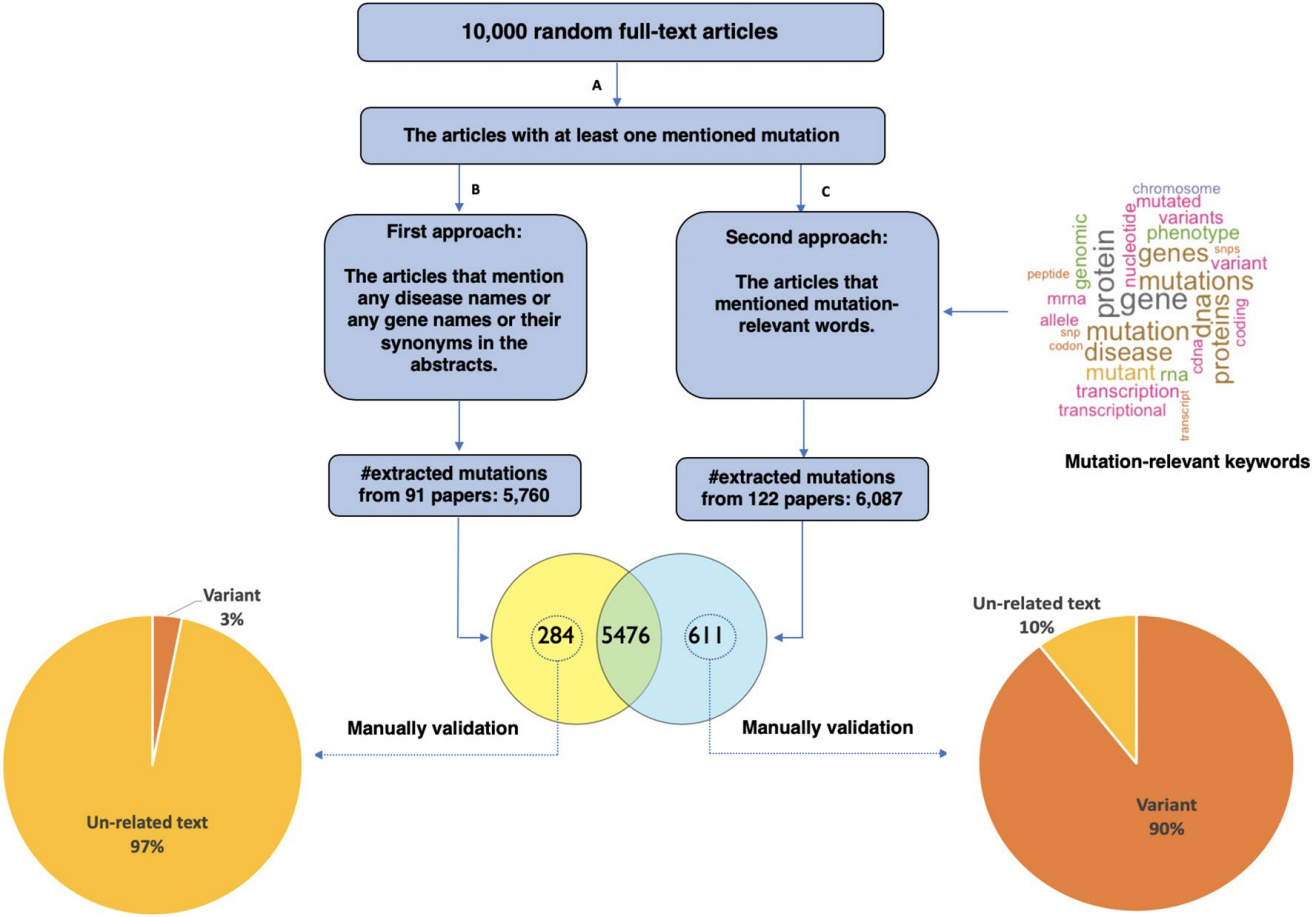
Among the 10,000 random articles, the articles with at least one mentioned mutation are selected (using tmVar 2.0). We compare the performances of two different approaches for detecting the variant-relevant articles. The first approach identifies articles that mention any disease or gene or any of their synonym in their titles and abstracts. In the second approach, we only search for the articles that mention the variant-relevant keywords in their full-body text. The variant-relevant keywords is a weighted list of the words that appear frequently in a set of 10,000 random articles with at least one mentioned variants (using tmVar 2.0). Subsequently, an article is considered to be relevant to variants if at least 10% of these variant-relevant keywords are appear in the full-body text. The number of variants that are found in the articles selected by the first approach and the second approach are 5,760 and 6,087, respectively. The number of variants identified by both approaches is 5,476. The number of variants that are only found by the first approach is 284, of which 97% are false positive (unrelated text wrongly identified as a variant). The number of variants that are only found by the second approach is 611, of which only 10% are false positive. These results show that the second approach which is based on the variant-relevant keywords outperforms the first approach.

The input of the proposed framework consists of 3,322,746 full-length articles downloaded from the PMC database on January 2020. The variant indexing procedure from a full-length article is challenging because any chemical formulae, figure numbers, etc. that are represented in “Character-Number-Character” format could be identified as a variant^21^. One solution to address this challenge is to mine only the variant-relevant articles. We compare the performances of two different approaches for detecting the variant-relevant articles. The first approach considers only the articles that mention any disease or gene or any of their synonyms in the title and abstract sections. In the second approach, we employ the word cloud analysis and generate a weighted list of variant-relevant keywords. In particular, we first generate a weighted list of words (referred to as variant-relevant keywords) that appear frequently in the full-body text of 10,000 random articles with at least one mentioned variant (using tmVar 2.0). Subsequently, an article is considered to be relevant to variants if at least 10% of these keywords appear in the full-body of the article. We apply both approaches on a set of 10,000 random full-length articles. Figure 2 shows the identified variant overlaps and differences between the two approaches. The number of papers with at least one mentioned variant overlapped between the two approaches is 836 and the number of overlapped variants is 5,476. The number of variants that are only found by the first approach is 284 from 91 papers, in which a manual validation process revealed that 97% of them are false positive (unrelated text wrongly identified as a variant). The number of variants that are only found by the second approach is 611 from 122 papers, in which only 10% of them are false positive. These results show that the second approach which is based on the variant-relevant keywords outperforms the first approach. The manual validation of the extracted variants are included in the Supplementary materials (Table S1). This leads us to the conclusion that the second approach performs better in terms of the ability to index the variant-relevant articles. This approach results in a list of 1,274,775 full-length articles that contain genomic variants.

### Extract the variant, gene and disease entities

We use the publicly available and well-known entity recognition tools to extract the variant, gene and disease phenotype from each input article. In particular, we use GNormPlus^27^ to identify the appropriate genes. The tmVar 2.0^21^ is the tool we employ for extracting the variants and normalizing those which are included in dbSNP to their unique identifiers (dbSNP RSIDs). We use DNorm^28^ to identify all the disease phenotypes mentioned in an article.

### Extract the variant-gene associations

Once a variant is extracted from an input article, we follow the steps provided by Wei et al.^21^ to find the associated gene. Then, we map each retrieved variant-gene pair to the corresponding genomic coordinates (chromosome number, position, reference and alternative alleles) using the Variant Recoder^30^ tool. We eliminate the variant-gene pairs with no matched genomic coordinates (referred to as false positive pairs).

### Extract the variant-disease associations

We use a set of features to capture the variant-disease associations from an input article adapted from the method proposed by Singhal et al.^1^. Let $C= \{ {V, D_{\text {1}}, D_{\mathrm {2}} ,\ldots, D_{\text {k}}} \}$ be a collection of appearances of the variant *V* and the closest (based on the word counts) mentioned diseases in an article, where *k* is the number of times this variant is mentioned in that article. The disease association score is calculated for each appearance of variant *V* and the closest mentioned disease *D*$_{\text {i}}$, where $1\le \hbox {i}\le \hbox {k}$. This score is the summation of the following set of scores:

- The Same Sentence Occurrence (SSO) is a binary score which is 1 when the variant *V* and the disease $D_{\text {i}}$ are mentioned in the same sentence and 0 otherwise.
- The Same Paragraph Occurrence (SPO) is a binary score which is 1 when the variant *V* and the disease $D_{\text {i}}$ are mentioned in the same paragraph and 0 otherwise.
- The sentiment score (SS) calculates the polarity sentiment value for the text mentioned between the variant *V* and the disease $D_{\text {i}}$. We use the R package “sentimentr”^31^ for this analysis.

The variant *V* is considered to be associated with disease $D_{\text {i}}$ that has the highest disease association score.

We also perform an experiment to compare the performance of the proposed scoring method for extracting the variant-disease associations with the simple sentence co-occurrence scoring method (baseline method). In this experiment, we use the two manually curated benchmark datasets provided by Doughty et al.^16^. These datasets contains variant-disease pairs extracted from 29 and 129 PubMed articles for prostate cancer and breast cancer, respectively. We use these datasets and report the standard evaluation metrics (precision, recall and F1 score) for both methods. As shown in Table 1, the proposed method outperforms the baseline method. The complete list of mined variant-disease pairs for this experience are included in the Supplementary Materials (Table S2).

**Table 1:** Comparison of the proposed variant-disease association scoring method with the baseline approach (co-occurrence only) on the benchmark datasets.

Corpus	Evaluation metrics	Proposed method	Baseline method
Breact cancer	Precison	0.90385	0.31731
	Recall	0.85455	0.30000
	F1 measure	0.87850	0.30841
Prostate cancer	Precison	0.91111	0.37778
	Recall	0.85417	0.35417
	F1 measure	0.88172	0.36559

These datasets are provided by Doughty et al.^16^. The proposed approach performs better compare to the baseline approach.

### Variant-driven gene panel design

In this step, we first generate a variant-gene-disease panel which includes all the associations between the gene, variant and disease entities extracted from the input corpus (Module D in Fig. 1). This panel includes 18,254 genes with 313,780 variants discovered to be associated with 5,202 unique diseases. For a given disease, we then generate the variant-driven gene panel which includes all the genes with at least one mentioned variant discovered to be associated with the given disease.

### Validation

**Figure 3:**
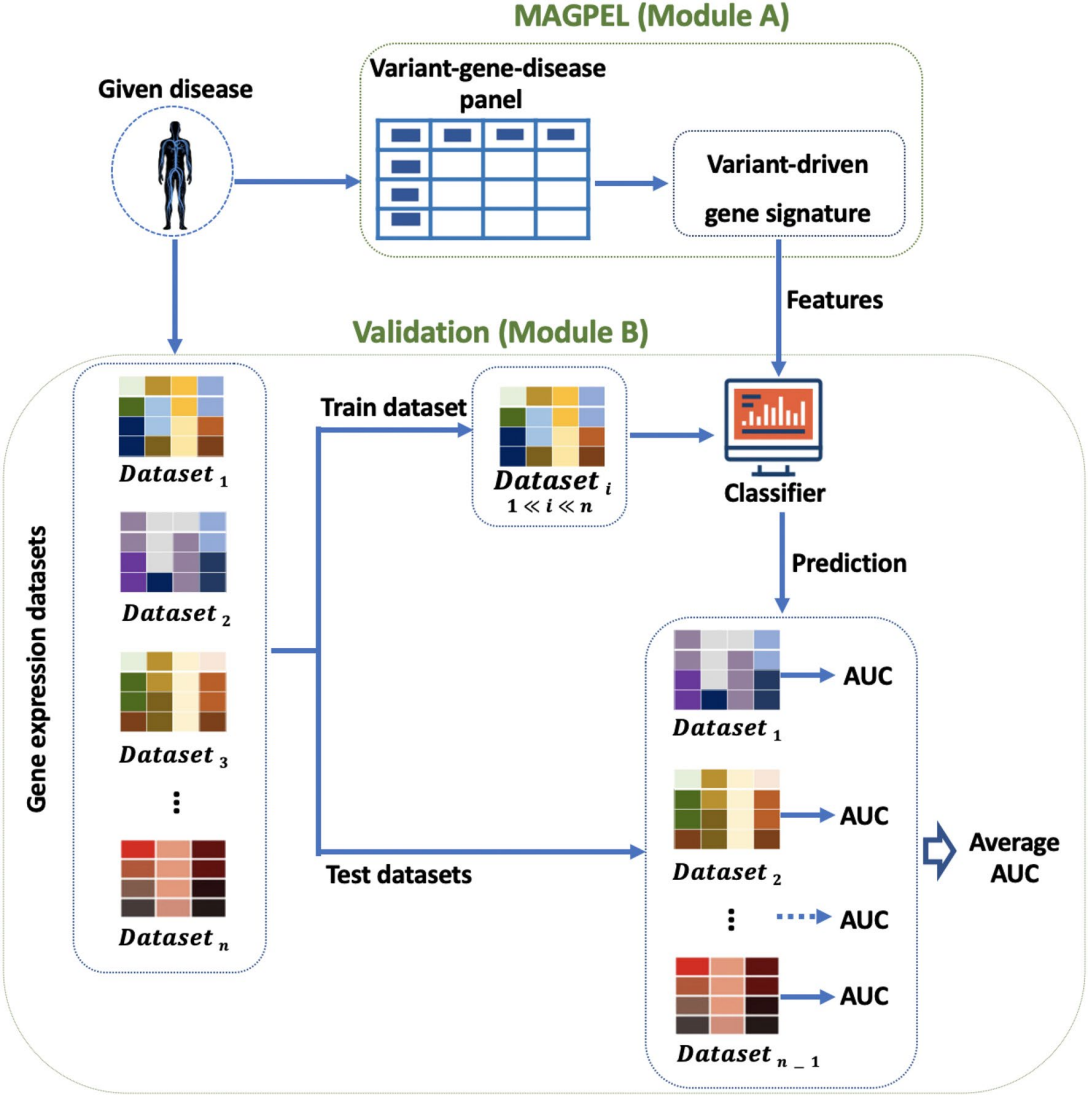
Validation framework overview. Module (**A**) identifies all the genes with at least one variant discovered to be associated with the given disease by the proposed framework. We refer to this list of genes as the proposed variant-driven gene panel. Module (**B**) first analyzes several independent gene expression datasets studying the given phenotype. We use cross validation method. In each round of sampling, we use one of the gene expression datasets as the training dataset and we use the rest as the testing datasets. We use the expression values of the genes included in the proposed gene panel as the features to build a classifier. Then, we apply the trained classifier on each of the testing datasets in order to predict the patients’ clinical outcome in each testing dataset. We use the area under the curve (AUC) of the receiver-operator characteristic to assess the performance of the classifier. We repeat this procedure *n* times (where *n* is the number of gene expression datasets). An average of AUCs is calculated over the *n* rounds of sampling. This procedure is used to compare the diagnostic quality of the proposed variant-driven gene panel with the current available variant-relevant gene panels obtained from literature.

In this section we describe the two experiments performed to assess the diagnostic value of the proposed variant-driven gene panels. In the first experiment, we use the genes present in the proposed panel to predict the patients’ clinical condition (healthy vs. disease) from several independent patient cohorts (Fig. 3). The hypothesis is that a better gene panel will yield better classification results. For this purpose, we use disease gene expression datasets and machine-learning classification techniques. A disease gene expression dataset is a matrix in which the rows represent the measured genes and the columns represent the samples (healthy or disease individual). The value in each cell is the expression level of a gene in a particular sample. We use cross validation method for this analysis. In particular, in each round of sampling, we use one of the gene expression datasets as the training dataset and we use the rest as the testing datasets. We use the genes present in the proposed variant-driven gene panel along with their expression values from the training dataset to build a random forest classifier^32^. Then, we apply the trained classifier on each of the testing datasets in order to predict the patients’ clinical outcome. We use the area under the curve (AUC) of the receiver-operator characteristic (ROC) to assess the performance of the classifier. We repeat this procedure *n* times (where *n* is the number of available gene expression datasets). An average of the AUCs is calculated over the *n* rounds of sampling. This procedure is used to compare the diagnostic quality of the proposed gene panel with the current available variant-relevant gene panels obtained from literature.

In the second experiment, we assess the relevance of the proposed gene panel to the given disease based on the rank of target pathway when an enrichment pathway analysis is performed. For each signaling pathway, the enrichment pathway analysis method calculates the probability of finding a center number of gene overlaps between the proposed gene panel and the presented genes in each pathway just by chance and then ranks the pathways based on this probability^33^. The detailed descriptions of the enrichment pathway analysis method are included in the Supplementary Materials. A “target pathway” refers to the pathway that was created to explain the mechanism of the given disease (e.g. the acute myeloid leukemia KEGG pathway (hsa05221) is the target pathway for acute myeloid leukemia). The expectation here is that a gene panel that is relevant to the given disease would rank the target pathway at the very top of the ranked list of pathways. This validation method was widely adopted by others, such as^34–42^. In addition, not only the target pathway but also the other identified significantly enriched pathways provide crucial information to assess the performance of the proposed gene panel. We also provide the top 10 significantly enriched pathways and the references explaining the association of the respective pathways to the disease case study for each gene panel in the Supplementary Materials.

## Results

As representative examples, we present the results for acute myeloid leukemia (AML), breast cancer and prostate cancer. The resulted gene panel proposed for each case study are included in the Supplementary Materials (Table S3). All the gene expression datasets used in this manuscript for the classification analysis are obtained from GEO^43^. Dataset summaries are described in the Supplementary Materials. For each disease case study, we also calculate the percentage of the genes in the proposed gene panel that overlap with the genes in each gene expression dataset. We perform this experiment as a quality check to ensure that the majority of the genes in the proposed gene panel are contributing to the validation analysis (Module B in Fig. 3). For each case study, the average of this percentage across all the gene expression datasets is more than 80%. The results and details of this experiment are included in the Supplementary Materials.

### Acute myeloid leukemia (AML)

First, we extract all the genes with at least one mentioned variant discovered to be associated with AML by the proposed framework. The top 10 genes that have the highest number of variants are TP53, FLT3, KIT, DNMT3A, IDH1, COX8A, RUNX1, TYMS, NPM1 and SLC29A1. These genes play significant roles in the underlying mechanism of AML. For instance, Kadia et al.^44^ demonstrated that the AML patients with TP53 alterations have lower response rate to the intensive chemotherapy and therefore have inferior survival rate. FLT3 and C-KIT are known to be associated with poor AML prognosis discovered by Pratz et al.^45^ and Yang et al.^46^, respectively. Ley et al.^47^ investigated the role of DNMT3A and found that there is a direct link between the presence of mutations in this gene and the intermediate risk of AML. Chaturvedi et al.^48^ also reported the therapeutic role of mutant IDH1 in AML. Gaidzik et al.^49^ have shown that the therapy-resistance and inferior outcomes are the main genetic characteristics of the AML patients with RUNX1 mutations. The presence of mutations in TYMS and NPM1 are also discovered in AML patients^50,51^. SLC29A1 mutations are also found to be associated with poor therapy outcome in AML patients^52^.

**Figure 4:**
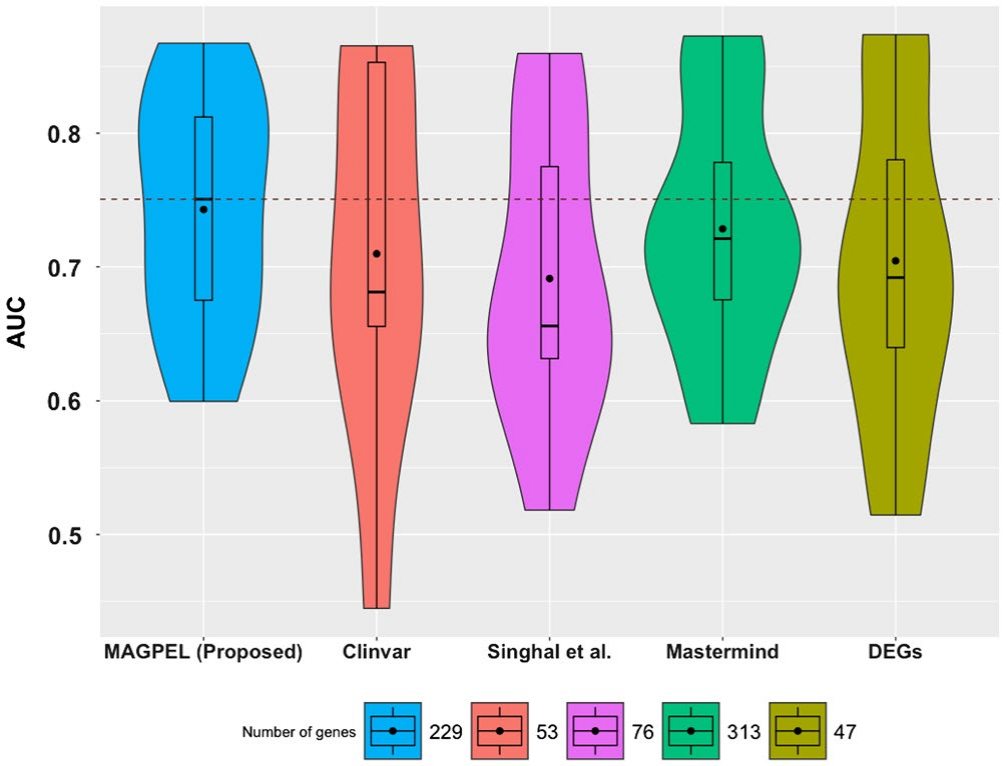
The diagnostic performances of the random forest classifier based on five different gene panels. In this figure, the proposed panel (blue panel) performs better than the ones obtained from Clinvar (red panel), Mastermind^29^ (green panel), the panel proposed by Singhal et al.^25^ (purple panel) and the differentially expressed genes (FDR-corrected *p*-value$<0.05$ and $|\log _{2}$(fold change)$|>=1.5$) (DEGs) (olive-tone panel) in terms of the ability to distinguish between healthy volunteers and the AML patients. In this figure, the black dot inside each box plot represents the mean AUC value and the dash line represents the highest median AUC value.

We assess the utility of the proposed gene panel on independent gene expression datasets studying AML obtained from GEO^43^. The other variant-driven gene panels which are available for AML are obtained from Clinvar^2^, Mastermind^29^ and the panel proposed by Singhal et al.^25^. Clinvar is a repository for mutations and their associated disease phenotypes which are manually curated from the biomedical literature. The Mastermind search engine provides literature-based variant-genotype-phenotype association information. We also include the results when using only the differentially expressed genes (FDR-corrected *p*-value $<0.05$ and $|\log _{2}$ fold change)$|>=1.5$) as a gene panel. Figure 4 illustrates the performance comparison of these gene panels. The results show that the classification based on the proposed gene panel achieves the best result (the highest median AUC value) and outperforms the classification based on all the other published panels.

### Prostate cancer

In this case study we discover 532 genes with variants associated to prostate cancer. The proposed prostate cancer variant-driven gene panel contains several genes known to be involved in prostate cancer development and progression. For instance, the androgen receptor (AR) plays important role in prostate cancer cell proliferation as demonstrated by Balk et al.^53^. The mutated BRCA2, TP53, KLK3 and RNASEL genes are directly associated with the risk of developing prostate cancer^54–57^. SPOP is also the most frequent mutated gene in the primary prostate cancer^58,59^.

**Figure 5:**
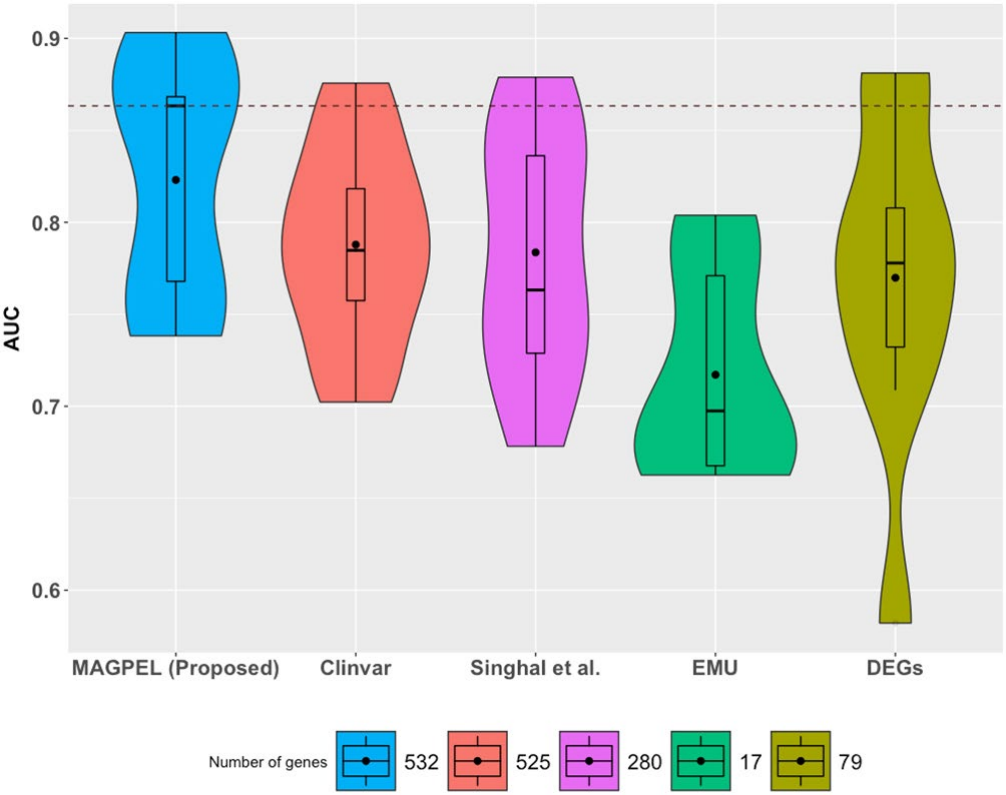
The diagnostic performances of the random forest classifier based on five different gene panels. In this figure, the proposed panel (blue panel) performs better than the ones obtained from Clinvar (red panel), the panels proposed by Singhal et al.^25^ (purple panel), EMU^16^ (green panel) and also the differentially expressed genes (FDR-corrected *p*-value $<0.05$ and $|\log _{2}$ (fold change)$|>=1.5$) (DEGs) (olive-tone panel) in terms of the ability to distinguish between healthy volunteers and the breast cancer patients. In this figure, the black dot inside each box plot represents the mean AUC value and the dash line represents the highest median AUC value.

The classification results also demonstrate that the proposed gene panel outperforms the other available gene panels^2,16,25^ in terms of the ability to predict the patients’ clinical outcome on several independent validation cohorts (Fig. 5).

### Breast cancer

The resulted panel for breast cancer includes 513 genes. This panel also contains several genes that are known to play crucial roles in the underlying mechanism of breast cancer. For instance, BRCA1, BRCA2, TP53, ESR1, PIK3CA, ERBB2 and PALB2 are among the genes with high number of variants associated to breast cancer. The mutations in BRCA1, BRCA2, and TP53 are well-known to be associated with a high breast cancer risk^60,61^. ESR1 mutations are involved in the hormone-resistant metastatic breast cancer^62–66^. PIK3CA is an oncogene in breast cancer^67–70^ and ERBB2 is shown to be up-regulated in several breast tumors^71–74^. PALB2 is also reported as one of the breast cancer susceptibility genes^75–78^.

**Figure 6:**
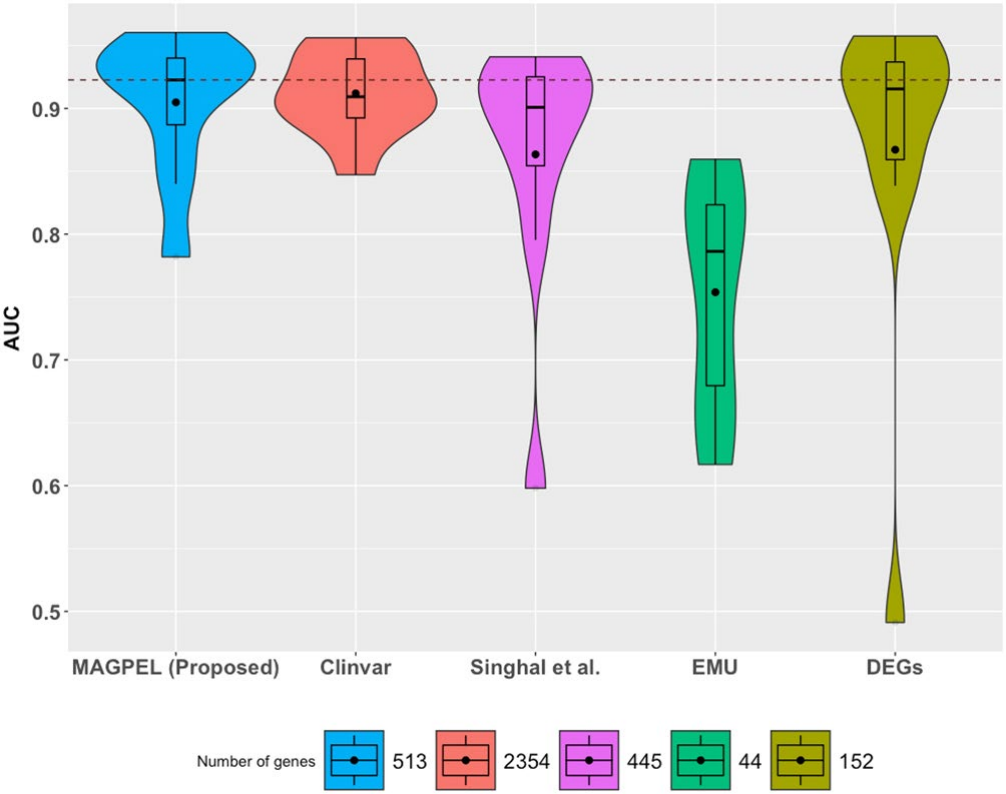
The diagnostic performances of the random forest classifier based on five different gene panels. In this figure, the proposed panel (blue panel) performs better than the ones obtained from Clinvar (red panel), the panels proposed by Singhal et al.^25^ (purple panel) and Doughty et al.^16^ (green panel) and also the differentially expressed genes (FDR-corrected *p*-value$<0.05$ and $|\log _{2}$(fold change)$|>=1.5$) (DEGs) (olive-tone panel) in terms of the ability to distinguish between healthy volunteers and the breast cancer patients. In this figure, the black dot inside each box plot represents the mean AUC value and the dash line represents the highest median AUC value.

We compare our panels with several other previously proposed variant-driven breast cancer gene panels as follows: i) Clinvar^2^, ii) Singhal et al.^25^, iii) Doughty et al.^16^ and iv) the classical DEGs. The classification results demonstrate that the gene panel proposed here performs better than the other gene panels in terms of the ability to predict the patients’ clinical outcome on several independent validation datasets (Fig. 6).

## Discussion

**Figure 7:**
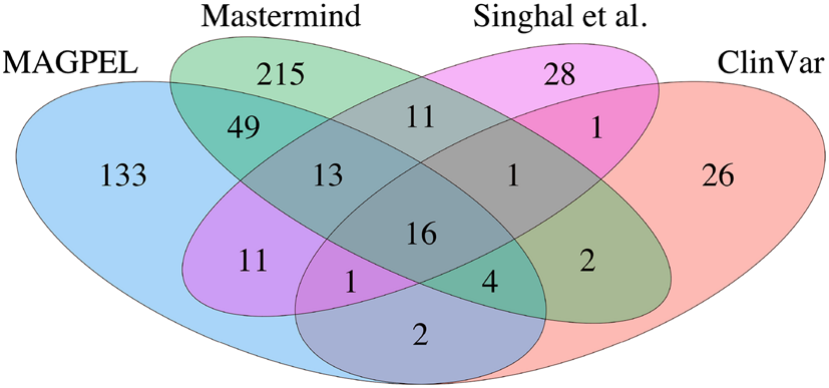
An overview of the gene overlaps and differences between the variant-driven gene panels. The proposed gene panel (MAGPEL) consists of 229 genes. The AML-related gene panel obtained from Clinvar and Mastermind includes 53 and 313 genes, respectively and the one proposed by Singhal et al.^25^ includes 76 genes.

**Figure 8:**
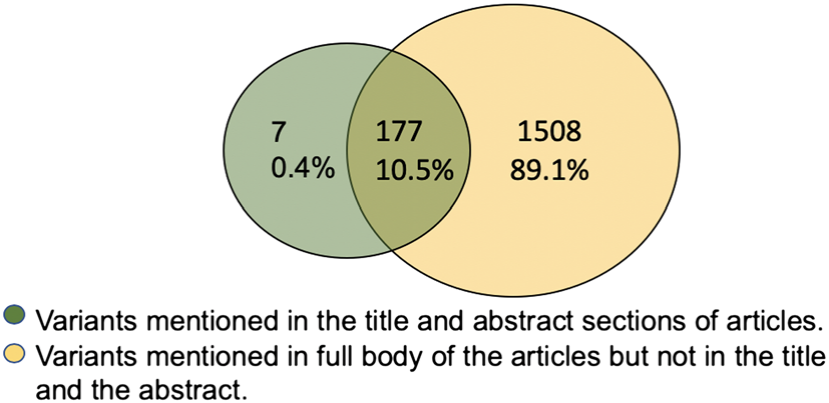
An overview of the overlap and differences between the variants mentioned in the title and abstract sections of the articles (green) and those that are appear in the full body of the articles but not in the title and abstract section (gold).

We investigate the novelty of our identified genes by checking their overlap with other available variant-driven gene panels for AML (Fig. 7). Although 58% of the proposed genes are not included in the other panels, the classification and pathway analysis based on these genes achieves the best results. The gene differences between the proposed panel and Clinvar could arise from the fact that Clinvar is a manually curated database. In principle, manual curation is expected to yield very accurate but possibly incomplete annotations, which is consistent with the smaller number of genes included in the Clinvar panel. The consideration of only the title and abstract of the articles for extracting the variants by Singhal et al.^25^, could be the reason for the gene differences between these two panels. We also investigate the percentage of the identified AML-related variants which are mentioned in the title and abstract sections of the articles, and compared them with those that are mentioned in the full body of the articles but not in the title and the abstract. Figure 8 visualizes the variant overlaps and differences between these sections. As the figure shows, about 89% of the variants mentioned in an article do not appear in the title and the abstract sections, which emphasizes the need to analyze the entire text of the articles. This represents a significant limitation of the existing methods that use only the title and abstract sections of an article for indexing variants. The venn diagrams for other case studies are included in the Supplementary Materials.

## Conclusion

The number of articles describing the disease-related variants is rapidly increasing. This highlights the pressing need for the development of automated tools that are able to extract the variant-disease associations from literature. In this article, we implement an automated framework to extract the variant-gene-disease associations from the full-length biomedical literature and design an evidence-based variant-driven gene panel for a given disease. The identification of the variant-relevant articles using word cloud analysis, and the consideration of the full-length articles are the main contributions of the proposed framework. We illustrate the utilities of the proposed variant-driven gene panels in capturing the mechanisms involved in AML, prostate cancer, and breast cancer using 27 independent gene expression datasets containing a total 2,109 patients. The results show that the proposed gene panel outperforms the other published gene panels in terms of the ability to predict the patients’ clinical outcome.

## Electronic supplementary material

Supplementary information

Supplementary Table S1

Supplementary Table S2

Supplementary Table S3

## Data Availability

The proposed variant-driven gene panels are available as part of the Supplementary Materials. The datasets generated and analyzed during the current study are available from the corresponding author upon request.
