# Supplementary material for: MAGPEL: an autoMated pipeline for inferring vAriant-driven Gene PanEls from the full-length biomedical literature
Source: Sci Rep. 2020 Jul 23;10:12365. doi: 10.1038/s41598-020-68649-0 (PMC7378213; doi:10.1038/s41598-020-68649-0)
Supplement: Supplementary file 1 — Supplementary information [file 41598_2020_68649_MOESM1_ESM.pdf]

MAGPEL: an autoMated pipeline for inferring vAriant-driven Gene PanEls from the full-length biomedical Literature

Supplementary Materials

Nafiseh Saberian[1], Adib Shafi[1], Azam Peyvandipour[1], and Sorin Draghici[1,2*]

[1]Department of Computer Science, Wayne State University, Detroit, MI 48202

[2]Department of Obstetrics and Gynecology, Wayne State University, Detroit, MI 48202

Enrichment Pathway Analysis

A signaling pathway refers to a graph in which nodes represent genes/proteins, and edges represent existing interactions between such genes or proteins. In general, the main goal of the pathway analysis methods is the correct identification of the pathways that are significantly impacted when comparing two phenotypes (*e.g.* healthy vs. disease) [9, 14]. Many pathway analysis methods have been proposed [18, 19, 14]. A very recent extensive benchmarking of the existing pathway analysis methods are provided by Nguyen *et al.* [20].

In this manuscript, we use the enrichment pathway analysis method called over-representation analysis (ORA) [13]. The goal of this method is to find the pathways that are enriched within a list of genes. In particular, this method calculates the probability of finding a center number of gene overlaps between the proposed gene panel and the presented genes in each pathway just by chance. For a pathway P, this probability is calculated as follows:

$$p\text{-}value = 1 - \sum_{i=0}^{k-1} \frac{\binom{M}{i}\binom{N-M}{n-i}}{\binom{N}{n}} \tag{1}$$

In this equation, N is the total number of genes in genome that have been annotated, n is the total number of genes in the proposed gene panel, k is the total number of gene overlaps between the proposed gene panel and the pathway P, and M is the total number of genes included in the pathway P.

These probability values are calculated for all pathways. Subsequently, they should be adjusted for multiple comparison with an approach such as false discovery rate correction (FDR) [4, 5]. For each pathway, if the FDR-corrected p-value is less than a certain threshold (usually less than 0.05), then the pathway is considered to be significantly involved in the experiment. For this analysis we use the R package "clusterProfiler v3.12.0" [27]. The list of significant pathways are then ranked from the one with the lowest FDR-corrected p-value (most significant) to the one with the highest p-value (least significant). The expectation is that the top ranked pathways are more involved in the given condition.

Tables 1 to 3 summarize the top 10 significantly enriched pathways and the references explaining the association of the respective pathways to the given disease for each gene panel.

Rank	Pathway name	p-value (FDR)
1	Acute myeloid leukemia	1.57E-15
2	PI3K-Akt signaling pathway [1, 16]	2.42E-11
3	Transcriptional misregulation in cancer [21]	1.44E-10
4	Prostate cancer	1.44E-10
5	ErbB signaling pathway [26]	1.44E-10
6	Chronic myeloid leukemia [22]	2.26E-10
7	PD-L1 expression and PD-1 checkpoint pathway in cancer	2.26E-10
8	JAK-STAT signaling pathway [10]	1.26E-09
9	Hepatitis B	1.26E-09
10	EGFR tyrosine kinase inhibitor resistance	3.18E-09

(a) Proposed gene panel (MAGPEL)

Rank	Pathway name	p-value (FDR)
1	PI3K-Akt signaling pathway [1]	2.53E-07
2	Acute myeloid leukemia	8.36E-07
3	Central carbon metabolism in cancer [6]	8.36E-07
4	PD-L1 expression and PD-1 checkpoint pathway in cancer	3.76E-06
5	Thyroid cancer	1.54E-05
6	Bladder cancer	1.89E-05
7	Chronic myeloid leukemia [22]	1.89E-05
8	EGFR tyrosine kinase inhibitor resistance	2.08E-05
9	Endometrial cancer	8.42E-05
10	Non-small cell lung cancer	0.00014

(b) Clinvar [17] gene panel

Rank	Pathway name	p-value (FDR)
1	JAK-STAT signaling pathway [10]	2.13E-17
2	Chronic myeloid leukemia [22]	4.70E-15
3	Acute myeloid leukemia	1.62E-14
4	Human T-cell leukemia virus 1 infection	1.44E-10
5	Transcriptional misregulation in cancer [21]	1.53E-09
6	Hepatitis B	2.39E-09
7	PI3K-Akt signaling pathway [1]	2.39E-09
8	Non-small cell lung cancer	4.48E-09
9	Central carbon metabolism in cancer [6]	6.29E-09
10	Pancreatic cancer	1.51E-08

(c) Singhal *et al.* [25] gene panel

Rank	Pathway name	p-value (FDR)
1	Hematopoietic cell lineage	6.98E-37
2	JAK-STAT signaling pathway [10]	1.11E-34
3	PI3K-Akt signaling pathway [1]	6.78E-34
4	Transcriptional misregulation in cancer[21]	1.97E-30
5	Hepatitis B	2.60E-30
6	Human T-cell leukemia virus 1 infection	8.14E-30
7	Epstein-Barr virus infection	7.66E-29
8	Kaposi sarcoma-associated herpesvirus infection	1.53E-27
9	Acute myeloid leukemia	5.50E-26
10	Prostate cancer	6.83E-26

(d) Mastermind [11] gene panel

Table 1: The top 10 significantly enriched pathways identified by the enrichment pathway analysis based on four different gene panels for AML case study. Rows with green background indicate the target pathway for AML. Rows with blue background indicate pathways for which we found indication of their association to AML.

Dataset Description

All the independent validation datasets are obtained from GEO [3]. Summary of the datasets used for AML, prostate cancer and breast cancer are provided in Tables 4 to 6.

Rank	Pathway name	p-value (FDR)
1	Prostate cancer	5.49E-28
2	FoxO signaling pathway [23]	3.13E-25
3	Endocrine resistance	2.29E-21
4	Colorectal cancer	2.29E-21
5	Pancreatic cancer	3.54E-21
6	PI3K-Akt signaling pathway [24]	2.35E-20
7	AGE-RAGE signaling pathway in diabetic complications	4.05E-19
8	Endometrial cancer	3.96E-18
9	Chronic myeloid leukemia	6.04E-18
10	Bladder cancer	1.51E-17

(a) Proposed gene panel (MAGPEL)

Rank	Pathway name	p-value (FDR)
1	Pancreatic cancer	1.93E-06
2	Endometrial cancer	1.96E-06
3	Melanoma	1.69E-05
4	Endocrine resistance	1.69E-05
5	Breast cancer	2.71E-05
6	Non-small cell lung cancer	2.74E-05
7	Prostate cancer	5.10E-05
8	Colorectal cancer	6.29E-05
9	Glioma	7.57E-05
10	Bladder cancer	8.38E-05

(b) Clinvar [17] gene panel

Rank	Pathway name	p-value (FDR)
1	Endometrial cancer	4.96E-08
2	Prostate cancer	5.83E-07
3	Gastric cancer	5.13E-06
4	Colorectal cancer	8.28E-06
5	Platinum drug resistance	0.00015237
6	Hepatocellular carcinoma	0.00015268
7	Thyroid cancer	0.00054001
8	Bladder cancer	0.00064535
9	Breast cancer	0.00133837
10	Hepatitis C	0.00147899

(c) EMU [8] gene panel

Rank	Pathway name	p-value (FDR)
1	Prostate cancer	8.12E-12
2	Hepatitis B	1.82E-08
3	Platinum drug resistance	2.85E-07
4	Bladder cancer	2.85E-07
5	FoxO signaling pathway [23]	3.12E-07
6	Steroid hormone biosynthesis	1.66E-06
7	Pancreatic cancer	1.95E-06
8	Transcriptional misregulation in cancer	1.95E-06
9	PI3K-Akt signaling pathway [24]	3.08E-06
10	Endometrial cancer	5.58E-06

(d) Singhal *et al.* [25] gene panel

Table 2: The top 10 significantly enriched pathways identified by the enrichment pathway analysis based on four different gene panels for prostate cancer case study. Rows with green background indicate the target pathway for prostate cancer. Rows with blue background indicate pathways for which we found indication of their association to prostate cancer.

Rank	Pathway name	p-value (FDR)
1	Proteoglycans in cancer	2.54E-27
2	Pancreatic cancer	2.41E-24
3	ErbB signaling pathway [12]	1.14E-23
4	Colorectal cancer	1.41E-23
5	Endocrine resistance	2.51E-21
6	Chronic myeloid leukemia	3.95E-21
7	Endometrial cancer	1.67E-20
8	Hepatitis B	1.46E-18
9	Prostate cancer	1.57E-18
10	EGFR tyrosine kinase inhibitor resistance	1.94E-18

(a) Proposed gene panel (MAGPEL)

Rank	Pathway name	p-value (FDR)
1	Herpes simplex virus 1 infection	2.67E-21
2	Taste transduction	0.000787
3	Natural killer cell mediated cytotoxicity [2]	0.000787
4	Antigen processing and presentation	0.00196105
5	Fanconi anemia pathway	0.00196105
6	B cell receptor signaling pathway	0.00317
7	Human papillomavirus infection	0.00691236
8	Graft-versus-host disease	0.00691236
9	Pancreatic secretion	0.02699356
10	Aldosterone-regulated sodium reabsorption	0.02707948

(b) Clinvar [17] gene panel

Rank	Pathway name	p-value (FDR)
1	Prostate cancer	8.68E-11
2	Endometrial cancer	3.85E-10
3	Homologous recombination [7]	6.27E-10
4	Melanoma	1.33E-09
5	Platinum drug resistance	1.33E-09
6	Breast cancer	1.46E-09
7	Hepatocellular carcinoma	5.35E-09
8	Bladder cancer	1.38E-08
9	Gastric cancer	2.44E-08
10	Glioma	2.74E-08

(c) EMU [8] gene panel

Rank	Pathway name	p-value (FDR)
1	Proteoglycans in cancer	1.01E-20
2	Colorectal cancer	2.49E-20
3	Pancreatic cancer	4.76E-20
4	Prostate cancer	7.23E-20
5	Chronic myeloid leukemia	4.46E-19
6	Gastric cancer	3.30E-18
7	ErbB signaling pathway [12]	1.22E-17
8	Hepatocellular carcinoma	2.89E-17
9	Endometrial cancer	6.50E-17
10	Endocrine resistance	6.76E-17

(d) Singhal *et al.* [25] gene panel

Table 3: The top 10 significantly enriched pathways identified by the enrichment pathway analysis based on four different gene panels for breast cancer case study. Rows with green background indicate the target pathway for breast cancer. The rank of target pathway obtained based on MAGPEL, Clinvar and Singhal *et al.* [25] is 12, 22 and 152, respectively. Rows with blue background indicate pathways for which we found indication of their association to breast cancer.

Dataset	Title	#Disease samples	#Control samples
GSE15061	Gene array prediction of AML transformation in MDS	202	69
GSE17054	Dysregulated gene expression networks in human acute myelogenous leukemia stem cells	9	4
GSE2191	pediatric AML and normal bone marrow	54	4
GSE34577	Routine use of microarray-based gene expression profiling to identify patients with low cytogenetic risk acute myeloid leukemia	21	8
GSE35008	Expression data from human hematopoietic stem and progenitor compartments from patients with acute myeloid leukemia with normal karyotype and healthy controls	12	16
GSE37307	Aberrant expressed genes in AML	30	19
GSE42140	Gene expression in signaling subsets of AML blasts induced by G-CSF	33	7
GSE9476	Abnormal expression changes in AML	26	38
GSE982	Gene Expression-Based High Throughput Screening: HL-60 Cell Treatment with Candidate Compounds	9	6

Table 4: Summary of the datasets used for AML case study.

Dataset	Title	#Disease samples	#Control samples
GSE12348	Prostate cancer cell lines and normal prostate epithelial and stromal cells in primary culture	6	3
GSE17906	Gene expression down-regulation in prostate tumor-associated stromal cells involves organ-specific genes	10	10
GSE17951	Gene expression analysis of prostate cancer samples using Affymetrix U133Plus2 array	68	13
GSE32448	CPDR tumor-benign 80 genechip dataset	40	40
GSE46602	Expression data from prostate cancer and benign prostate glands	36	14
GSE55945	Gene expression profiling of prostate benign and malignant tissue	13	8
GSE68882	Comprehensive gene expression analysis of prostate cancer reveals distinct transcriptional programs associated with metastatic disease	23	3
GSE6956	Tumor immunobiological differences in prostate cancer between african-american and european-american men	69	18
GSE70768	Prostate cancer stratification using molecular profiles	125	74

Table 5: Summary of the datasets used for prostate cancer case study.

Dataset	Title	#Disease samples	#Control samples
GSE10780	Proliferative genes dominate malignancy-risk gene panel in histologically-normal breast tissue	42	143
GSE10810	Gene expression panels in breast cancer distinguish phenotype charact., histological subtypes, and tumor invasivness	31	27
GSE20086	Heterogeneity of gene expression in stromal fibroblasts of human breast carcinomas and normal breast	6	6
GSE29431	Identifying breast cancer biomarkers	25	12
GSE36295	Transcriptomic analysis of breast cancer	45	5
GSE42568	Breast cancer gene expression analysis	67	17
GSE54002	Gene expression profiling of LCM captured breast cancer cells	417	16
GSE61304	Novel bio-marker discovery for stratification and prognosis of breast cancer patients	56	4
GSE86374	Analysis of somatic DNA copy number alterations and frequency of breast cancer intrinsic subtypes from Mexican women	50	36
GSE8977	Bone-marrow-derived mesenchymal stem cells promote breast cancer metastasis	7	15

Table 6: Summary of the datasets used for breast cancer case study.

Results

For each disease case study, we calculate the percentage of the genes in the proposed gene panel that overlap with the genes in each gene expression dataset. We perform the following experiment as a quality check to ensure that the majority of the genes in the proposed gene panel are contributing to the validation analysis. In order to do this, we calculate the percentage of the genes in the proposed gene panel that overlap with the genes in the classification training dataset as follows:

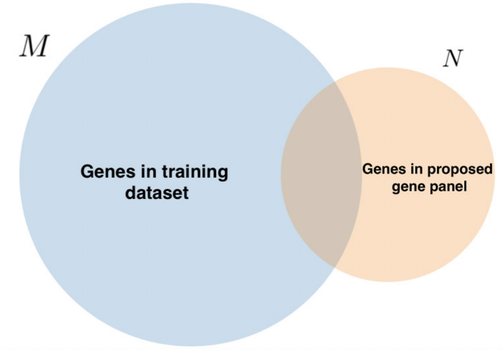

$$P = \frac{|N \cap M|}{|N|}$$

In this equation, N represents the genes in the proposed gene panel and M represents the genes in the training gene expression dataset. For each case study, the average of this percentage across all the gene expression datasets is more than 80% (Tables 7 to 9).

Discussion

Mastermind is a genomic search engine which is not publicly available. So, we had no choice but to use the published Mastermind variant-driven gene panel which is available for AML but not for prostate cancer and breast cancer [15]. We use the published EMU [8] gene panels which are available for breast cancer and prostate cancer, but not for AML.

The proposed automated framework will be implemented as part of an advanced web-based analysis tool named iVariantGuide (`https://advaitabio.com/ivariantguide/`) which will be updated on a regular basis.

Dataset	MAGPEL (proposed)	Mastermind [15]	Clinvar [17]	Singhal *et al.* [25]
GSE15061	96.94	97.44	88.68	90.24
GSE17054	96.51	97.12	88.68	90.24
GSE34577	97.38	98.4	88.68	90.24
GSE35008	92.58	95.53	86.79	87.8
GSE37307	90.39	95.21	79.25	84.15
GSE42140	96.51	97.12	88.68	90.24
GSE9476	90.39	95.21	79.25	84.15
GSE982	90.39	95.21	79.25	84.15
Average	93.88	96.40	84.90	87.65

Table 7: The percentage of the genes in each AML gene panel that overlap with the genes in each GEO gene expression dataset.

Dataset	MAGPEL (proposed)	EMU [8]	Clinvar [17]	Singhal *et al.* [25]
GSE12348	86.28	100.00	68.01	85.87
GSE17906	97.18	100.00	89.08	94.35
GSE17951	97.18	100.00	89.08	94.35
GSE32448	97.18	100.00	89.08	94.35
GSE46602	97.18	100.00	89.08	94.35
GSE55945	97.18	100.00	89.08	94.35
GSE68882	75.56	82.35	50.00	72.44
GSE6956	86.28	100.00	68.01	85.87
GSE70768	98.31	100.00	94.44	94.35
Average	92.48	98.04	80.65	90.03

Table 8: The percentage of the genes in each prostate gene panel that overlap with the genes in each GEO gene expression dataset.

Dataset	MAGPEL (proposed)	EMU [8]	Clinvar [17]	Singhal *et al.* [25]
GSE10780	97.66	100.00	85.64	86.90
GSE10810	75.05	72.73	50.21	61.90
GSE20086	97.66	100.00	85.64	86.90
GSE29431	97.66	100.00	85.64	86.90
GSE36295	94.74	95.45	85.47	85.69
GSE42568	97.66	100.00	85.64	86.90
GSE54002	97.66	100.00	85.64	86.90
GSE61304	97.66	100.00	85.64	86.90
GSE86374	94.74	95.45	85.47	85.69
GSE8977	92.01	95.45	73.83	79.44
Average	94.25	95.91	80.88	83.41

Table 9: The percentage of the genes in each breast cancer gene panel that overlap with the genes in each GEO gene expression dataset.

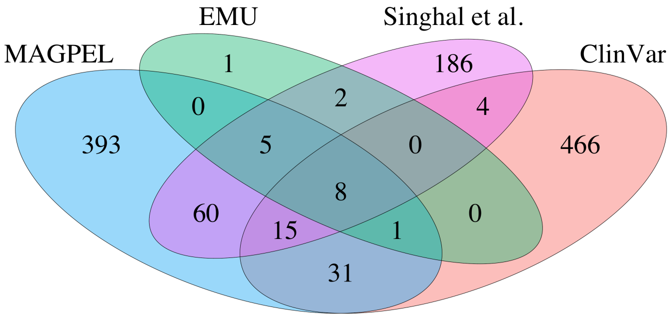

Figure 1: An overview of the gene overlaps and differences between the variant-driven gene panels for prostate cancer. The proposed gene panel (MAGPEL) consists of 532 genes. The prostate cancer-related gene panel obtained from Clinvar and EMU includes 525 and 17 genes, respectively and the one proposed by Singhal *et al.* [25] includes 280 genes.

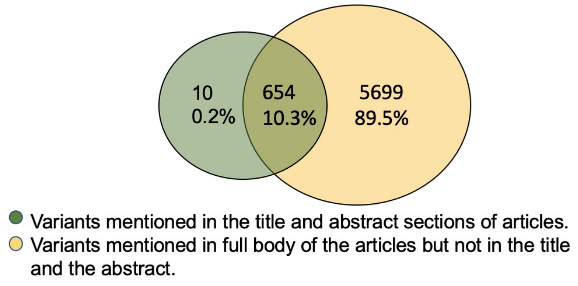

Figure 2: An overview of the overlap and differences between the variants mentioned in the title and abstract sections of the articles (green) and those that are appear in the full body of the articles but not in the title and abstract section (gold) in prostate cancer case study.

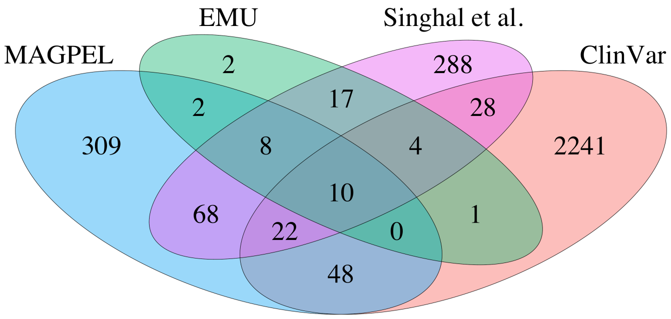

Figure 3: An overview of the gene overlaps and differences between the variant-driven gene panels for breast cancer. The proposed gene panel (MAGPEL) consists of 513 genes. The breast cancer-related gene panel obtained from Clinvar and EMU includes 2,354 and 44 genes, respectively and the one proposed by Singhal *et al.* [25] includes 445 genes.

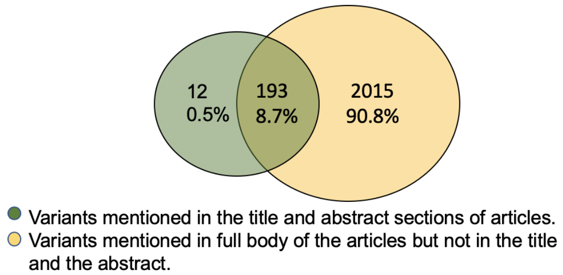

Figure 4: An overview of the overlap and differences between the variants mentioned in the title and abstract sections of the articles (green) and those that are appear in the full body of the articles but not in the title and abstract section (gold) in breast cancer case study.

References

[1] Yudi Ari Adi, Fajar Adi-Kusumo, Lina Aryati, and Mardiah S Hardianti. A dynamic model of pi3k/akt pathways in acute myeloid leukemia. *Journal of Applied Mathematics*, 2018, 2018.

[2] E Ames, WHD Hallett, and William J Murphy. Sensitization of human breast cancer cells to natural killer cell-mediated cytotoxicity by proteasome inhibition. *Clinical & Experimental Immunology*, 155(3):504–513, 2009.

[3] T. Barrett, T. O. Suzek, D. B. Troup, S. E. Wilhite, W. C. Ngau, P. Ledoux, D. Rudnev, A. E. Lash, W. Fujibuchi, and R. Edgar. NCBI GEO: mining millions of expression profiles–database and tools. *Nucleic Acids Research*, 33(Database Issue):D562–6, 2005.

[4] Y. Benjamini and Y. Hochberg. Controlling the false discovery rate: A practical and powerful approach to multiple testing. *Journal of The Royal Statistical Society B*, 57(1):289–300, 1995.

[5] Y. Benjamini and D. Yekutieli. The control of the false discovery rate in multiple testing under dependency. *Annals of Statistics*, 29(4):1165–1188, August 2001.

[6] Dominique Bonnet and John E Dick. Human acute myeloid leukemia is organized as a hierarchy that originates from a primitive hematopoietic cell. *Nature Medicine*, 3(7):730–737, 1997.

[7] Rémi Buisson, Anne-Marie Dion-Côté, Yan Coulombe, Hélène Launay, Hong Cai, Alicja Z Stasiak, Andrzej Stasiak, Bing Xia, and Jean-Yves Masson. Cooperation of breast cancer proteins palb2 and piccolo brca2 in stimulating homologous recombination. *Nature Structural & Molecular Biology*, 17(10):1247, 2010.

[8] Emily Doughty, Attila Kertesz-Farkas, Olivier Bodenreider, Gary Thompson, Asa Adadey, Thomas Peterson, and Maricel G Kann. Toward an automatic method for extracting cancer-and other disease-related point mutations from the biomedical literature. *Bioinformatics*, 27(3):408–415, 2010.

[9] Sorin Draghici. *Statistics and Data Analysis for Microarrays using R and Bioconductor*. Chapman and Hall/CRC Press, 2011.

[10] Stefan Faderl, Alessandra Ferrajoli, David Harris, Quin Van, Hagop M Kantarjian, and Zeev Estrov. Atiprimod blocks phosphorylation of jak-stat and inhibits proliferation of acute myeloid leukemia (aml) cells. *Leukemia Research*, 31(1):91–95, 2007.

[11] Genomenon. Mastermind: Automated gene panel design mobilizing evidence from the medical literature. https://mastermind.genomenon.com, 2017.

[12] Katharine M Hardy, Brian W Booth, Mary JC Hendrix, David S Salomon, and Luigi Strizzi. Erbb/egf signaling and emt in mammary development and breast cancer. *Journal of Mammary Gland Biology and Neoplasia*, 15(2):191–199, 2010.

[13] Purvesh Khatri and Sorin Draghici. Ontological analysis of gene expression data: current tools, limitations, and open problems. *Bioinformatics*, 21(18):3587–3595, 2005.

[14] Purvesh Khatri, Marina Sirota, and Atul J Butte. Ten years of pathway analysis: current approaches and outstanding challenges. *PLOS Computational Biology*, 8(2):e1002375, 2012.

[15] Mark Kiel, Lauren Chunn, Diane Nefcy, Ryan Tarpey, and Stephanie Wisner. Mastermind: Mobilizing evidence from the medical literature. https://www.genomenon.com.

[16] Y Kubota, H Ohnishi, A Kitanaka, T Ishida, and T Tanaka. Constitutive activation of pi3k is involved in the spontaneous proliferation of primary acute myeloid leukemia cells: direct evidence of pi3k activation. *Leukemia*, 18(8):1438–1440, 2004.

[17] Melissa J Landrum, Jennifer M Lee, George R Riley, Wonhee Jang, Wendy S Rubinstein, Deanna M Church, and Donna R Maglott. Clinvar: public archive of relationships among sequence variation and human phenotype. *Nucleic Acids Research*, 42(D1):D980–D985, 2013.

[18] Cristina Mitrea, Zeinab Taghavi, Behzad Bokanizad, Samer Hanoudi, Rebecca Tagett, Michele Donato, Călin Voichiţa, and Sorin Draghici. Methods and approaches in the topology-based analysis of biological pathways. *Frontiers in Physiology*, 4:278, 2013.

[19] Tin Nguyen, Cristina Mitrea, and Sorin Draghici. Network-based approaches for pathway level analysis. *Current Protocols in Bioinformatics*, 61(1):8–25, 2018.

[20] Tuan-Minh Nguyen, Adib Shafi, Tin Nguyen, and Sorin Draghici. Identifying significantly impacted pathways: a comprehensive review and assessment. *Genome Biology*, 20(1):1–15, 2019.

[21] Raeuf Roushangar and George I Mias. Multi-study reanalysis of 2,213 acute myeloid leukemia patients reveals age-and sex-dependent gene expression signatures. *Scientific Reports*, 9(1):1–17, 2019.

[22] Charles L Sawyers. Chronic myeloid leukemia. *New England Journal of Medicine*, 340(17):1330–1340, 1999.

[23] Zhengfei Shan, Yongwei Li, Shengqiang Yu, Jitao Wu, Chengjun Zhang, Yue Ma, Guimin Zhuang, Jiantao Wang, Zhenli Gao, and Dongfu Liu. Ctcf regulates the foxo signaling pathway to affect the progression of prostate cancer. *Journal of Cellular and Molecular Medicine*, 23(5):3130–3139, 2019.

[24] Sanjeev Shukla, Gregory T MacLennan, Douglas J Hartman, Pingfu Fu, Martin I Resnick, and Sanjay Gupta. Activation of pi3k-akt signaling pathway promotes prostate cancer cell invasion. *International Journal of Cancer*, 121(7):1424–1432, 2007.

[25] Ayush Singhal, Michael Simmons, and Zhiyong Lu. Text mining genotype-phenotype relationships from biomedical literature for database curation and precision medicine. *PLoS Computational Biology*, 12(11):e1005017, 2016.

[26] Melanie L Ufkin, Sarah Peterson, Xuehui Yang, Heather Driscoll, Christine Duarte, and Pradeep Sathyanarayana. mir-125a regulates cell cycle, proliferation, and apoptosis by targeting the erbb pathway in acute myeloid leukemia. *Leukemia Research*, 38(3):402–410, 2014.

[27] Guangchuang Yu, Li-Gen Wang, Yanyan Han, and Qing-Yu He. Clusterprofiler: an r package for comparing biological themes among gene clusters. *Omics: a Journal of Integrative Biology*, 16(5):284–287, 2012.
